# Degradation-Independent Inhibition of APOBEC3G by the HIV-1 Vif Protein

**DOI:** 10.3390/v13040617

**Published:** 2021-04-03

**Authors:** Benjamin Stupfler, Cédric Verriez, Sarah Gallois-Montbrun, Roland Marquet, Jean-Christophe Paillart

**Affiliations:** 1 CNRS, Architecture et Réactivité de l’ARN, UPR 9002, IBMC-2 Allée Konrad Roentgen, Université de Strasbourg, F-67000 Strasbourg, France; b.stupfler@ibmc-cnrs.unistra.fr (B.S.); c.verriez@ibmc-cnrs.unistra.fr (C.V.); r.marquet@ibmc-cnrs.unistra.fr (R.M.); 2 Sorbonne Paris Cité, INSERM U1016, CNRS UMR8104, Institut Cochin-27 rue du Faubourg Saint-Jacques, Université Paris Descartes, F-75014 Paris, France; sarah.gallois-montbrun@inserm.fr

**Keywords:** HIV, Vif, APOBEC3G, ubiquitin, proteasome, translation, encapsidation, deamination, RNP granules

## Abstract

The ubiquitin–proteasome system plays an important role in the cell under normal physiological conditions but also during viral infections. Indeed, many auxiliary proteins from the (HIV-1) divert this system to its own advantage, notably to induce the degradation of cellular restriction factors. For instance, the HIV-1 viral infectivity factor (Vif) has been shown to specifically counteract several cellular deaminases belonging to the apolipoprotein B mRNA-editing enzyme catalytic polypeptide-like (APOBEC3 or A3) family (A3A to A3H) by recruiting an E3-ubiquitin ligase complex and inducing their polyubiquitination and degradation through the proteasome. Although this pathway has been extensively characterized so far, Vif has also been shown to impede A3s through degradation-independent processes, but research on this matter remains limited. In this review, we describe our current knowledge regarding the degradation-independent inhibition of A3s, and A3G in particular, by the HIV-1 Vif protein, the molecular mechanisms involved, and highlight important properties of this small viral protein.

## 1. Introduction

Once the human immunodeficiency virus type 1 (HIV-1) has entered the cell, it must overcome, or disarm, a network of restriction factors (including A3 enzymes) that block specific steps of the replication cycle [1], and hijack the intracellular machinery to its own benefit. Restriction factors play an important role in innate immune responses and protect the host from viral pathogens [1,2,3]. Members of the apolipoprotein B mRNA-editing enzyme catalytic polypeptide-like (APOBEC3 or A3) family are innate immune effectors restricting many exogenous viruses, such as HIV-1, endogenous retroelements [4,5,6,7], and several non-retroviral viruses [8]. The human genome encodes seven A3 genes (A3A, B, C, D, F, G, and H) resulting from gene duplications and rearrangements during mammalian evolution [9,10,11]. A3G was the first to be discovered and is the most functionally characterized enzyme. A3G was demonstrated to inhibit HIV-1 replication in the absence of the viral infectivity factor (Vif) [12] thanks to its cytidine deaminase activity that converts cytidine (C) to uridine (U) present in single-stranded DNA generated during reverse transcription of the viral genome. Completion of proviral DNA synthesis results in guanine (G) to adenine (A) mutations in the plus strand DNA, eventually leading to disruption of protein synthesis (through nonsense and/or missense mutations) and interruption of HIV-1 replication [13,14,15,16]. Amongst the seven A3 proteins expressed in human cells, A3D, F, and H have also been identified as anti-HIV-1 factors [17]. While the deaminase activity is essential for the A3 antiviral function, it has been reported that A3s could repress HIV-1 replication in a deaminase-independent manner at different stages of the replication cycle (reverse transcription, integration, and maturation) [8,18,19,20,21].

To counteract restriction factors’ antiviral activities, HIV-1 expresses several accessory proteins, and Vif was shown to efficiently degrade A3G in virus-producing cells and inhibit its incorporation into nascent viral particles [12,14,15,22,23]. This process requires the recruitment of an E3-ubiquitin ligase complex by Vif, leading to the proteasomal degradation of A3G [12,24,25,26]. Interestingly, the Vif–A3 interplay is the consequence of an evolutionary arms race between lentiviruses and their hosts [27,28], i.e., interactions involving Vif and A3 proteins are species-specific and not all A3s are submitted to proteasomal degradation and are capable of inhibiting viral replication. For instance, A3B does not restrict HIV-1 and is not degraded by the HIV-1 Vif protein [29,30], at least for some HIV-1 Vif isolates (IIIB, JR-CSF, 89.6), while Vif from HIV-1 HXB2, ELI-1, and YU-2 shows some degradative effects [31]. SIV Vif proteins behave similarly [29], SIVmac239 Vif being the most potent at inhibiting A3B through the canonical polyubiquitination mechanism [29]. Although the degradation-dependent mechanism has been extensively studied and documented in the literature for the last twenty years [12,14,24,25,26,32], degradation-independent mechanisms mediated by Vif could also account for the reduced levels of A3 proteins in cells and in viral particles. Our review mainly focuses on the latter mechanism, highlighting their importance to overcome the potent antiviral activity of A3s in general, and of A3G in particular. We will also describe processes developed by other viruses which do not encode a functional Vif protein to overcome A3 enzymatic activity.

## 2. Degradation-Dependent Inhibition of APOBEC3G by Vif: A Brief Overview

### 2.1. The Players: APOBEC3G and Vif

**Figure 1:**
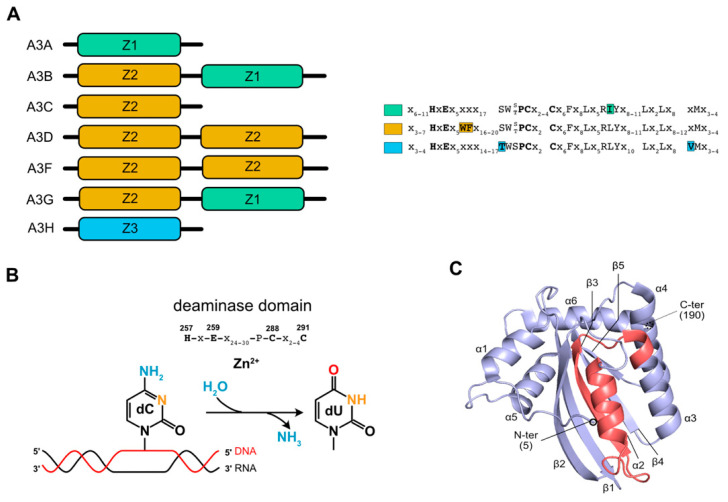
APOBEC3 structural and catalytic features. (**A**) Schematic structural domains of A3 proteins are represented. Characteristic phylogenetic ZDD domains are represented in color, with their amino acid sequences on the side. (**B**) A3G catalytic mechanism allowing cytidine deamination on a single-stranded DNA target (red strand) is represented. Amino acids constituting the catalytic site are mentioned above. These amino acids accommodate a zinc ion and the use of a water molecule leading to the deamination of the cytidine. (**C**) A3C crystal structure (hA3C) (PDB: 3VOW) represented in ribbons. Catalytic (CD) and ZDD domains are represented in purple and pink, respectively. Secondary structure motifs are annotated.

The seven members of the A3 family of proteins contain one (A3A, A3C, and A3H) or two (A3B, A3D, A3F, and A3G) copies of a catalytic deaminase (CD) domain that is distinguished by the presence of a signature motif, His-X-Glu-X_23-28_-Pro-Cys-X_2-4_-Cys (Figure 1A). When the enzyme contains two CDs, CD2 is catalytically active [33] while CD1 is involved in nucleic acids binding [34,35,36], its oligomerization and movements along the substrate to increase the deamination efficiency [37,38,39]. CDs and adjacent domains are organized into three phylogenetically distinct groups, named zinc-coordinating domains, Z1, Z2, and Z3 [11,40]. The mechanism of catalysis involves the histidine and the two cysteine residues that coordinate a zinc ion that enables the glutamic acid to deprotonate a water molecule, and generates the zinc hydroxide ion that will react with the carbon C4 of the cytosine (Figure 1B). The CDs’ tridimensional structure is conserved between A3s and is composed of five *β* strands and six *α* helices organized around the deaminase domain (Figure 1C) (for more details regarding the structure of A3 proteins, please refer to these recent reviews [33,41,42]).

Amongst A3 proteins, human A3G was the first to be identified and is the most potent inhibitor of HIV-1 replication in the absence of Vif protein [12], whereas A3D, A3F, and A3H (haplotype II, V, VII) have been shown to exhibit various degrees of antiviral activities [17,43]. A3D, A3F, A3G, and A3H are localized in the cytoplasm, A3A and A3C are distributed throughout the cell, while A3B is mainly nuclear [17,44]. When non-permissive cells are infected with HIV-1 ∆Vif viruses, A3G is efficiently incorporated into budding virions, a fundamental aspect of its antiviral function. A3G packaging is determined by specific interactions between its N-terminal CD domain and the nucleocapsid region of the HIV-1 Pr55^Gag^ precursor [45,46,47,48,49,50], but also depends on its association to RNAs, mainly the HIV-1 genomic and 7SL RNAs [35,36,49,50,51,52,53]. Following its packaging, A3G induces dC-to-dU deamination in the (−) strand DNA of HIV-1 during reverse transcription, resulting in G-to-A substitutions in the (+) strand DNA and eventually blocking viral replication due to the incorporation of missense or stop-codons [14,15,54].

The local nucleotide sequence preference for deamination is 5’-CC-3’ (the substrate C is underlined) for A3G and 5’-TC-3’ for other A3s [13,17,55,56]. In addition to G-to-A hypermutation, deaminase-independent activities could also contribute to the overall antiviral activity of A3G [18,21]. Indeed, A3G may bind to viral RNA and sterically interfere with the progression of reverse transcription [19,57,58,59] or bind the reverse transcriptase itself [60].

**Figure 2:**
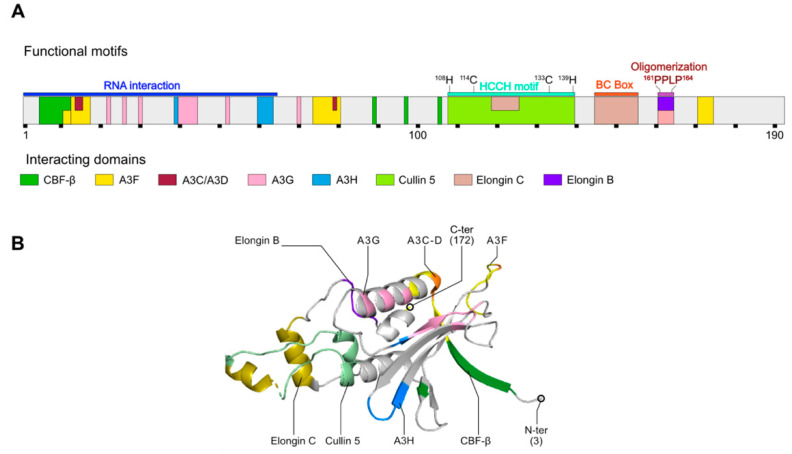
HIV-1 Vif protein structure and interactions with E3-ubiquitin ligase complex members. (**A**) Vif mono-dimensional structure (Genbank M19921; UniProtKB—P12504 (Vif_HV1N5)) is represented using 10 amino acids per scaling unit (black squares under the structure). Functional motifs are represented with colored rectangles above the structure. Interaction regions with A3 proteins or members of the E3-ubiquitin ligase are represented in color inside the structure. (**B**) Vif 3D structure (PDB: 4N9F; positions 3-172) is represented in ribbons. N- and C- terminal extremities (N-ter and C-ter, respectively) as well as interaction regions with several partners are colored and annotated on the structure. Adapted from [81].

HIV-1 overcomes A3G restriction by expressing Vif, which targets A3G for polyubiquitination and proteasomal degradation (see below) [12,24,25,26]. Initially considered as an accessory protein, Vif is in fact an important virulence factor whose absence considerably reduces viral infectivity [28,61]. Vif is a small, unstructured, and multimeric protein of 23 kDa expressed from singly-spliced viral mRNAs during the last stages of HIV-1 infection [62,63,64,65]. Notably, an intronic G run (G_I3-2_) has been shown to be critical for the splicing regulation of Vif mRNA in non-permissive cells in order to maintain an appropriate ratio of Vif to A3G protein levels [66,67,68], but this ratio is highly dependent on the physiological environment. Vif is mainly localized in the cytoplasm of infected cells but is also found in viral particles [69,70,71,72,73]. It possesses various properties linked to its functional domains and RNA chaperone activities (Figure 2): for instance, Vif was shown to influence HIV-1 particle morphology, maturation of Pr55^Gag^, reverse transcription, and HIV-1 genomic RNA dimerization [68,74,75,76,77,78]. However, one of the principal functions of Vif is to induce the polyubiquitination and subsequent proteasomal degradation of A3G, thereby reducing the pool of cytosolic A3G available for incorporation into viral particles [25,79,80]. 

### 2.2. Recruitment of an Ubiquitin Ligase Complex by Vif

**Figure 3:**
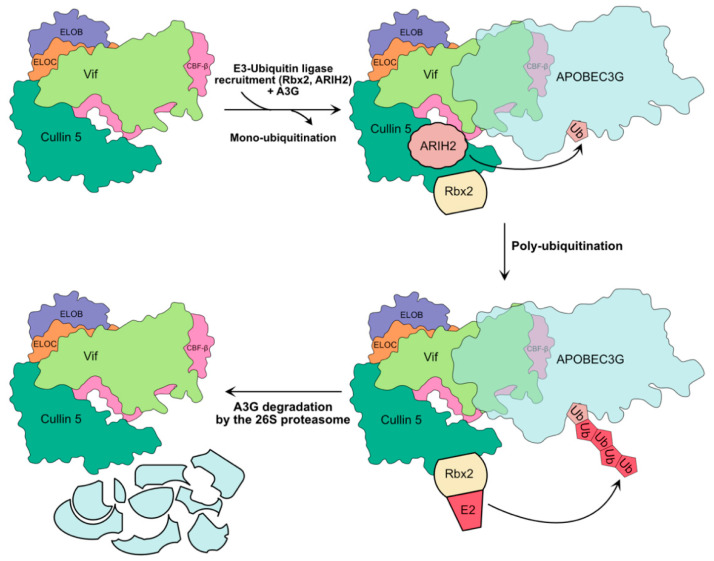
Hijacking of ubiquitin–proteasome pathway by Vif. HIV-1 first recruits the core components of the E3-ubiquitin ligase complex, namely Elongin B (ELOB) and ELOC, CBF-β, and Cullin 5. This complex is represented in accordance with its known 3D structure (PDB: 4N9F). The recruitment of A3G and catalytic subunits Rbx2 and ARIH2 leads to ARIH-2 mediated mono-ubiquitination of A3G. Subsequent recruitment of an E2 ubiquitin-conjugating enzyme by RBX2 leads to the poly-ubiquitination of A3G and its degradation by the 26S proteasome.

The most studied mechanism allowing the restriction of A3G is the recruitment by Vif of an E3-ubiquitin ligase complex [25,26,82], which is composed of the scaffold protein Cullin 5, Elongin B/C adaptor proteins, RBX2 catalytic units (RING-box protein 2), transcription factor CBF-*β* (Core-Binding Factor *β*), which plays a critical role in stabilizing Vif and its assembly with the ligase, and ARIH2 (Ariadne homolog 2) [24,25,26,32,83,84] (Figure 3). This last partner has been recently identified to act in an E1-E2-E3/E3 cascade to target A3G for degradation and has been shown to be essential for efficient HIV-1 infection in primary CD4+ T cells [83]. This allows the ^48^K poly-ubiquitinylation of A3G and its redirection towards the proteasome, which ensures its degradation. Many studies have mapped the domains and peptide sequences involved in the interaction between Vif, A3G, and the ligase complex (Figure 2, and for recent reviews on these different domains see [33,41,81]). The crystal structure of the human Vif-EloB/C-Cul5-CBF-*β* (PDB: 4N9F) [85] and simian Vif-EloB/C-CBF-*β* (PDB: 6P59) [86] have been solved and allowed to validate most of the protein–protein interactions previously determined by mutagenesis and biochemical assays. Very recently, a complex structure of the CBF-*β*-fused A3F_CTD_ with HIV-1 Vif has been obtained by cryo-EM and this structure may provide a structural insight into A3-Vif interaction [87], although further investigations are required to understand their interaction.

## 3. Degradation-Independent Inhibition of APOBEC3G by Vif

Although the primary mechanism of A3G neutralization in HIV-1 infected cells involves its ubiquitination and subsequent degradation by the 26S proteasome, other mechanisms have been shown to reduce A3G packaging into viral particles (through a direct or an indirect effect of Vif) or to interfere with the deaminase activity of encapsidated A3G. The following section will focus on these different mechanisms.

### 3.1. Transcriptional Inhibition of APOBEC3G, Hijacking of CBF-β by Vif

**Figure 4:**
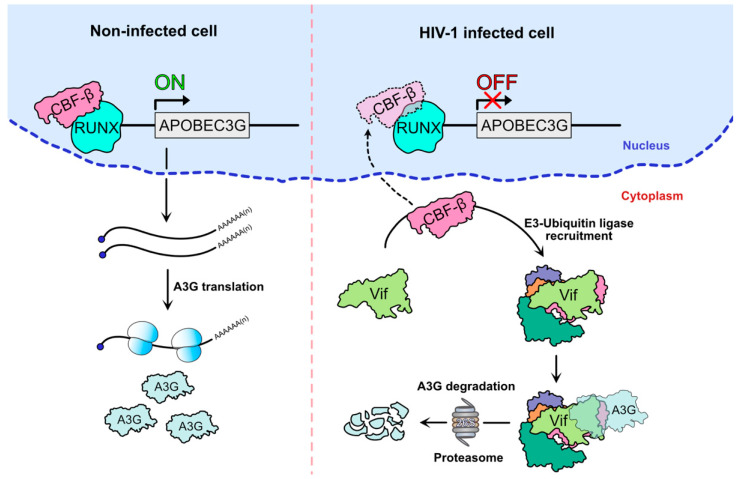
APOBEC3G mRNA transcriptional inhibition after CBF-β recruitment by HIV-1 Vif. The binding of CBF-*β* to the RUNX complex drives the transcription of the A3G gene and maintains a robust antiviral state in the absence of HIV-1 infection (**left**). In the presence of Vif (**right**), Vif hijacks CBF-*β* and prevents its binding to RUNX, thus simultaneously repressing A3G transcription and promoting its ubiquitination and proteasomal degradation.

The recruitment by Vif of the E3-ubiquitin ligase complex mediating the degradation of A3G may have an additional indirect effect on A3G expression. Indeed, the recruitment of CBF-*β* is mandatory for Vif stability and for the assembly of the E3-ubiquitin ligase complex responsible for A3G and other A3 proteins’ degradation [32,84]. The interaction was detected by mass spectrometry after the co-immunoprecipitation of tagged Vif construct in HEK 293 and Jurkat cells [32]. CBF-*β* is a non-DNA binding subunit that heterodimerizes with Runt-related transcription factor (RUNX) proteins to form the CBF family of transcription factors, which are important for cell differentiation and proliferation, hematopoiesis, and bone development [88]. Interestingly, the binding of Vif to CBF-*β* is mutually exclusive with RUNX heterodimerization and has been shown to impact the expression of genes whose regulatory domains are associated with RUNX1, which includes A3 genes, suggesting that Vif inhibits transcription by competing with RUNX for CBF-*β* binding [89,90] (Figure 4). Mutational analysis [91,92,93,94] and structural study of the pentameric E3-ubiquitin ligase complex (Vif-EloB/C-Cul5-CBF-*β*) [85] identified several Vif residues important for its binding to CBF-*β*. For instance, the N-terminal _5_WQVMIVW_11_, present in an anti-parallel *β*-strand of Vif, is a major interaction domain (Figure 2), along with residues W_89_, T_96_, and L_106_. Thus, the sequestration of CBF-*β* by Vif in the cytoplasm provides a dual hijacking mechanism by reducing A3G expression at both transcriptional and post-translational (degradation of A3G through the proteasome) levels.

### 3.2. Translational Inhibition of APOBEC3G mRNA by Vif

**Figure 5:**
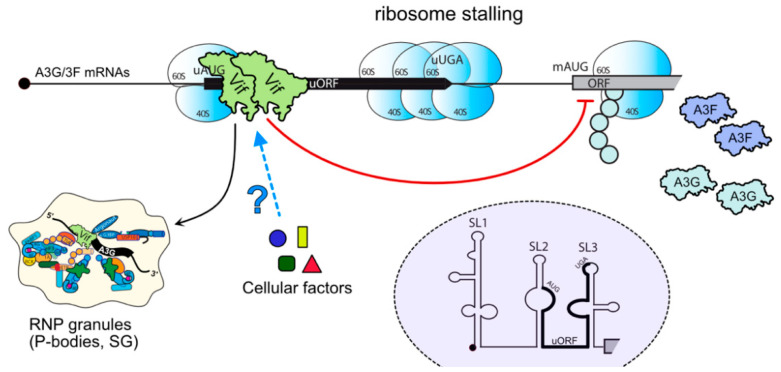
APOBEC3G mRNA translation inhibition mediated by HIV-1 Vif. Vif associates with the 5’UTR of A3G mRNAs, in the vicinity of A3G mRNA uORF. This association leads to A3G mRNA translation inhibition, possibly by ribosome stalling, and its relocation to RNP granules (P-bodies, stress granules, etc.). A3F mRNA could be regulated similarly as their 5’UTRs are phylogenetically conserved [102]. The involvement of other cellular factors in this process is still unknown but it could favor, or inhibit, A3G translation. The secondary structure of the 5’UTR of A3G mRNA as well as the position of the uORF (within SL2–SL3) are highlighted (purple dashed circle).

A second mechanism suggesting that Vif could reduce the intracellular level of A3G by affecting its translation was proposed shortly after the discovery of A3G [79,95]. First, the use of proteasome inhibitors on H9 infected cells or HEK 293 cells transiently co-transfected with Vif and A3G-HA expression vectors rescued only partial A3G expression, without altering the A3G mRNA levels. Second, ex-vivo pulse-chase experiments showed that Vif impaired the radiolabeling of the A3G-HA protein in a dose-dependent manner, reducing A3G translation by 30–40% [79,95]. Third, in vitro-coupled transcription/translation assays in rabbit reticulocyte lysates confirmed that Vif impaired A3G translation by approximatively 70–75% [79]. However, these first studies were performed using expression vectors lacking the authentic 5′ and 3′ untranslated regions (UTRs) of A3G mRNA, which could play a key role in A3G translation [96,97]. Thus, they may not faithfully recapitulate events occurring with endogenous A3G mRNA. Interestingly, we showed that Vif could bind A3G mRNA, and more specifically its UTRs [98]. Within the 5’UTR, two stem-loop structures (SL2–SL3) (Figure 5) were required for Vif to inhibit A3G translation [98,99] and viruses produced after transfection with expression vectors of A3G mRNAs lacking this motif showed reduced infectivity, which was mainly due to an increase in A3G encapsidation into viral particles [99]. Moreover, our results show that translational inhibition of A3G by Vif is independent of the proteasomal degradation pathway as some mutations in the N-terminus of Vif impede translation inhibition without affecting A3G degradation (K26R, this mutant still binds A3G [100]), while others present a complete inhibition of A3G degradation and translational reduction (H42/43N, this mutant is defective in A3G binding [101]), raising the possibility that Vif/A3G interaction might be required for translational regulation [99]. 

While mechanisms leading to A3G translation inhibition still remain unclear, we recently uncovered the importance of a short and conserved upstream ORF (uORF) located within SL2–SL3 of the 5’UTR of A3G and A3F mRNAs (Figure 5) [102]. Interestingly, the uORF represses A3G translation itself (40%), as do many uORF located in 5’UTR of mRNAs [103,104,105], but it is also mandatory for the Vif-mediated repression of A3G translation and for the redirection of A3G mRNA into stress granules in the presence of Vif [102]. The fact that Vif was previously shown in vitro to interact with the lower and upper stems of SL3 [98] suggests that Vif may block ribosome scanning (stalling). Given the proportion of genes that have one or more uORFs in their 5’UTR [103,104,105], one cannot rule out the possibility that Vif might be able to regulate the translation or the trafficking of such mRNAs in the cell. Notably, A3G was shown by immunofluorescence studies to localize to P-bodies and stress granules [31,106,107,108,109,110]; on the contrary, Vif was observed in P-bodies only in the presence of A3G, suggesting that Vif could selectively remove A3G from and/or restrict its localization to P-bodies to induce its degradation [107].

Taken together, translational inhibition of A3G by Vif seems to be a multi-layer process, involving direct (ribosome stalling at the 5’UTR) and indirect (shuttling of A3G mRNA to ribonucleoprotein granules) blockages to delay or prevent mRNA translation. Whereas additional experiments will be needed to clearly decipher this mechanism, one can imagine that Vif interacts with components of the eukaryotic translation initiation machinery to reduce the translation rate of A3G and/or recruit cellular factors involved in the negative regulation of the translation (Figure 5).

### 3.3. Packaging Inhibition of APOBEC3G by Vif

Packaging of A3G/3F proteins into HIV-1 particles depends on the nucleocapsid (NC) domain of Pr55^Gag^, and the current view is that its packaging is also reliant upon its capacity to bind RNA [35,45,46,47,48,50,53,111]. A3C has evolved to use distinct mechanisms for retrovirus targeting by interacting with the matrix (MA) domain of Pr55^Gag^ for its encapsidation [112]. A3D–H have been shown to be efficiently packaged into viral particles [17] and a correlation exists between the multimerization state of these A3s and their packaging (and restrictive) capacities [113,114]. Several studies have suggested that Vif prevents A3G packaging into HIV-1 particles independently of the reduction in its intracellular concentration [22,25,95,115]. Indeed, comparison of wild-type and ∆vif HIV-1 virions produced from transfected cells expressing a similar level of A3G showed that wild-type virions incorporated less A3G than ∆vif virions, suggesting that Vif can directly exclude A3G from progeny virions [25,95]. Moreover, A3G exclusion requires biologically active Vif. Indeed, the Vif C_114_ and C_133_ are critical residues for Cullin 5 interaction, as the HCCH motif and for Vif-mediated A3G degradation. Interestingly, the mutation of these residues (C_114_/S and C_133_/S) impaired Vif’s ability to inhibit A3G packaging [95]. Additional experiments further highlighted that A3G packaging inhibition was not linked to its Vif-induced intracellular degradation. In fact, an A3G C_97_/A mutant, which was defective for multimerization but resistant to Vif-mediated degradation, showed a decreased packaging yield in the presence of Vif [113,116]. Likewise, a recent study using antibody antigen-binding fragments (Fabs) directed against the ubiquitin transfer reaction and Vif-E3 assembly showed that the ubiquitination of A3C, A3F, and A3G was inhibited, although not sufficiently to restore their packaging into viral particles and antiviral activity [117]. These studies suggest that the inhibition of A3G packaging and A3G degradation are two distinct properties of Vif. Although a clear mechanism has not been described yet, it has been proposed that the RNA-binding capacities of A3G and Vif could account for this packaging restriction. Indeed, A3G and Vif share similar RNA-binding domains on HIV-1 genomic RNA, such as stem-loops in the 5’UTR packaging signal [51,71,118], and one can imagine that at early stages of assembly, Vif and A3G compete for their own packaging through HIV-1 RNA-binding, or that Vif prevents A3G encapsidation by masking a domain on A3G that mediates the interaction with the assembling virion [63]. As previously mentioned, it is well-established that Vif and A3G could be encapsidated through their capacity to bind the nucleocapsid (NC) domain of Pr55^Gag^ [45,46,47,111,119,120,121]. It is therefore conceivable that Vif may interfere with these A3G–Gag interactions. Notably, this inhibition may also favor reverse transcription as A3G was reported to reduce tRNA annealing to the primer binding site (PBS), which is required for the initiation of reverse transcription [122,123]. Interestingly, A3 (F/G/H) evolved to partially mimic the RNA-binding specificity of the HIV-1 NC (by targeting G-rich and A-rich sequences) in order to ensure their concomitant packaging with RNA into nascent virions [53]. Similar to a potential competition between Vif and A3G, it is conceivable that Gag may compete with A3 proteins for their encapsidation; thus, Gag–RNA occlusion could be seen as a countermeasure against A3s by retroviruses lacking a Vif protein. Alternatively, Vif could direct A3G away from sites of virus assembly by inducing the formation of the high molecular weight form of A3G [124,125], thus sequestering A3G in packaging-incompetent complexes.

### 3.4. Inhibition of Intravirion Deaminase Activity by Vif

While downregulation of A3G by Vif within the cell is very efficient (see above), A3G can still be found in viral particles during wild-type HIV-1 infection, even though to a lesser extent than in ∆vif viruses (4-11 A3G/∆vif(−) virion versus < 1 A3G/wild-type virion) [115,126,127,128,129]; thus, only a few A3G molecules are sufficient to potently inhibit HIV-1 replication. However, the efficiency of packaged A3G enzymes into wild-type particles indicated lower deaminase activity compared to those from ∆vif virions (65% reduction) [130], suggesting that virion-associated Vif molecules inhibit the intrinsic deaminase activity of A3G [131]. Indeed, earlier work performed in *Escherichia coli* showed that A3G-mediated hypermutation was inhibited by Vif [132], and this was correlated to a direct binding of Vif to A3G, as the use of a degradation-resistant A3G mutant (D128K, a species-specific determinant of Vif sensitivity) rendered A3G resistant to Vif inhibition [132,133]. In addition, enzymatic studies showed that Vif can attenuate the processivity of A3G by disrupting its scanning on viral ssDNA [133]. Taken together, these studies strongly argue in favor of a direct effect of Vif on A3G enzymatic activity, either through Vif–A3G binding (thus disrupting the binding of A3G to its substrate or A3G movements such as sliding or jumping), or by competing for its binding to the ssDNA substrate. Indeed, Vif is a well-known nucleic acids-binding protein [70,75,76,77,98,118,134,135,136]. Vif-binding sites that were identified in the 5’-end region of HIV-1 genomic RNA correspond to hypermutated sites in ∆vif virions [118], and Vif also binds with a significant affinity (Kd < 40 nM) to the consensus A3G ssDNA target sites [134]. Thus, Vif could interfere with A3G by masking its access to the neosynthesized viral DNA. Alternatively, in the target cell, it might locally limit the RNase H activity of HIV-1 reverse transcriptase that is necessary to allow the deaminase activity of A3G, which is otherwise inhibited upon binding to HIV-1 genomic RNA [125].

## 4. Degradation-Independent Inhibition of APOBEC3 Proteins by Other Viruses

The tumultuous relationship between A3s and viral proteins is also witnessed in other animal species and for various viruses. Indeed, viral proteins other than Vif have been shown to counteract A3 proteins in lentiviruses: this is the case for feline immunodeficiency virus (FIV) protease [137], HIV-1 protease (against A3H variant SV200) [138], and reverse transcriptase for A3G [139]. In other retroviruses, mechanisms developed to counteract A3 proteins have been adapted to compensate for the absence of a functional Vif-like gene. Foamy or spumaretroviruses are also restricted by A3 proteins, and these viruses use the accessory Bet protein, an equivalent of Vif, to inhibit A3 antiviral activity [140,141,142]. The interaction of feline foamy virus (FFV) and prototype/human foamy virus (PFV) Bet protein with feline A3 and A3F/3G proteins, respectively, reduces their packaging into viral particles without impacting their expression level [140,141]. Instead, Bet prevents A3G dimerization [143,144] and traps A3 (A3G/3C) in insoluble complexes, rendering them unavailable for virion packaging. Murine leukemia virus (MLV) possesses two mechanisms to overcome the restriction by mouse A3. On the one hand, its P50 protein, produced from an alternatively spliced *gag* RNA [145,146], prevents mouse A3 packaging by interacting with its C-terminus CD2 domain, but impacts neither its degradation nor its deaminase activity [147]. Considering that the C-terminus of mouse A3 is necessary for its incorporation into viruses, it is not surprising that P50 binding to this domain affects A3 packaging. On the other hand, glyco-Gag is known to be important for MLV-induced pathogenesis, and its abrogation leads to decreased virus replication and pathogenesis [148,149,150]. Glyco-Gag uses a unique mechanism to counteract the antiviral action of A3 by affecting the capsid stability and by protecting the reverse transcription complex in the viral cores from A3 proteins [151,152]. Human T-cell lymphotropic virus type 1 (HTLV-1) is also sensitive to human A3G, even though its cytidine deaminase activity is inoperant [153,154]. This virus has evolved a *cis*-acting mechanism to prevent A3G restriction. A peptide motif in the C-terminus of the NC domain inhibits A3G packaging into nascent virions [155]. In this case, the physical interaction between HTLV-1 NC and RNA would occlude A3G packaging. In addition, A3 proteins have been reported to inhibit several human pathogenic viruses other than retroviruses, such as the hepatitis B virus (HBV) [156], papillomavirus [157], and herpesvirus [158]. These viruses have evolved new strategies to antagonize the antiviral activities of A3s, either by enhancing the externalization of A3G by HBx (HBV), or by sequestering A3A/3B away from their site of activity thanks to the viral proteins BORF2 (Epstein–Barr virus, EBV), ICP6 (herpes simplex virus 1, HSV), and ORF6 (Kaposi’s sarcoma-associated herpesvirus) [159]. Notably, these viral proteins do not induce the degradation of A3 proteins.

## 5. Conclusions

**Figure 6:**
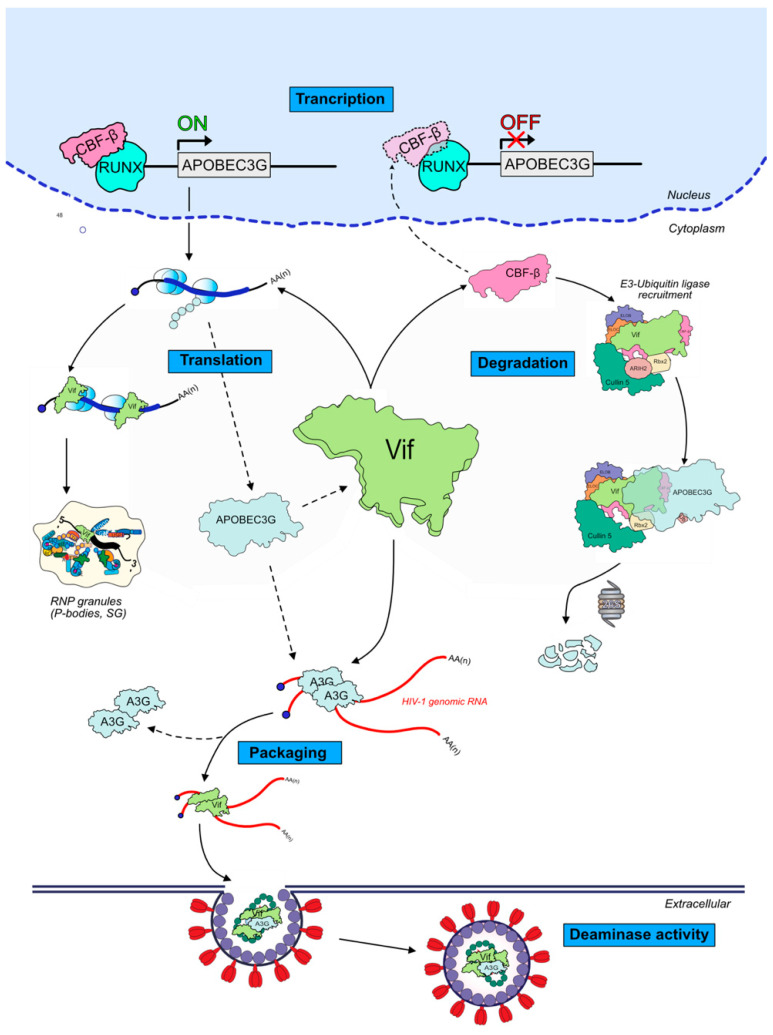
Summary of the different modes of inhibition of APOBEC3G by HIV-1 Vif protein. Other A3 proteins have also been shown to be regulated at different levels (see text for details): transcription (potentially all A3s); translation (A3G and A3F); degradation (all A3s, depending on considered HIV isolates and hosts); and packaging (A3D, A3F, A3G, and A3H).

The Vif protein is of major importance for an efficient viral infection in non-permissive cells by antagonizing A3 proteins antiviral activity. While mechanisms leading to A3G degradation through the recruitment of an E3-ubiquitin ligase complex by Vif are well understood, little is known concerning other degradation-independent routes used by Vif to inhibit A3G and other A3 proteins. These mechanisms are diverse, and aim to inhibit or reduce A3G transcription, translation, virion encapsidation, and enzymatic activity (Figure 6). Interestingly, Vif-proteins and Vif-RNA interactions are common characteristics between all these important steps, thus targeting them through their interacting interfaces may constitute promising antiviral strategies. A better knowledge of how the virus hijacks different cellular processes and which components are involved in viral replication is crucial in this attempt.
